# Advanced Glycation End Products (AGEs) May Be a Striking Link Between Modern Diet and Health

**DOI:** 10.3390/biom9120888

**Published:** 2019-12-17

**Authors:** Vidhu Gill, Vijay Kumar, Kritanjali Singh, Ashok Kumar, Jong-Joo Kim

**Affiliations:** 1 Central Research Station, Subharti Medical College, Swami Vivekanand Subharti University, Meerut 250002, India; vidhugill@gmail.com (V.G.); skritanjali@gmail.com (K.S.); 2 Department of Biotechnology, Yeungnam University, Gyeongsan, Gyeongbuk 38541, Korea; 3 Department of Medical Genetics, Sanjay Gandhi Post Graduate Institute of Medical Sciences, Lucknow 226014, India; chemistry.ashok83@gmail.com

**Keywords:** advanced glycation end products, Maillard reaction, diabetes, Alzheimer’s disease, polycystic ovarian syndrome, infant formula

## Abstract

The Maillard reaction is a simple but ubiquitous reaction that occurs both in vivo and ex vivo during the cooking or processing of foods under high-temperature conditions, such as baking, frying, or grilling. Glycation of proteins is a post-translational modification that forms temporary adducts, which, on further crosslinking and rearrangement, form permanent residues known as advanced glycation end products (AGEs). Cooking at high temperature results in various food products having high levels of AGEs. This review underlines the basis of AGE formation and their corresponding deleterious effects on the body. Glycated Maillard products have a direct association with the pathophysiology of some metabolic diseases, such as diabetes mellitus type 2 (DM2), acute renal failure (ARF), Alzheimer’s disease, dental health, allergies, and polycystic ovary syndrome (PCOS). The most glycated and structurally abundant protein is collagen, which acts as a marker for diabetes and aging, where decreased levels indicate reduced skin elasticity. In diabetes, high levels of AGEs are associated with carotid thickening, ischemic heart disease, uremic cardiomyopathy, and kidney failure. AGEs also mimic hormones or regulate/modify their receptor mechanisms at the DNA level. In women, a high AGE diet directly correlates with high levels of androgens, anti-Müllerian hormone, insulin, and androstenedione, promoting ovarian dysfunction and/or infertility. Vitamin D3 is well-associated with the pathogenesis of PCOS and modulates steroidogenesis. It also exhibits a protective mechanism against the harmful effects of AGEs. This review elucidates and summarizes the processing of infant formula milk and the associated health hazards. Formulated according to the nutritional requirements of the newborn as a substitute for mother’s milk, formula milk is a rich source of primary adducts, such as carboxy-methyl lysine, which render an infant prone to inflammation, dementia, food allergies, and other diseases. We therefore recommend that understanding this post-translational modification is the key to unlocking the mechanisms and physiology of various metabolic syndromes.

## 1. Introduction: A Brief Glance at Advanced Glycation End Products (AGEs)

**Figure 1:**
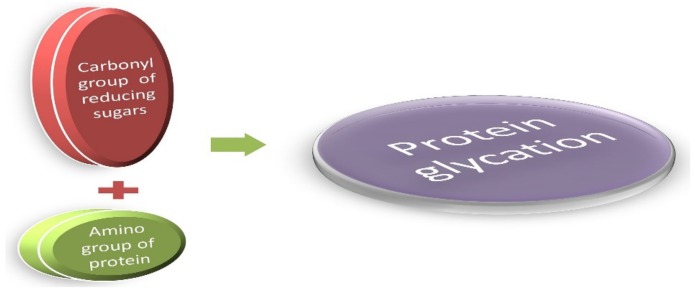
The Maillard reaction: A simple condensation reaction of protein and glucose. They are generally known as glycated proteins.

Cooking practices have evolved, along with the evolution of man, from eating raw meat to cooking on low flame and later modernized to high flame cooking, such as baking, caramelizing, or preserving meats with sugar and spices. These high-temperature cooking techniques, such as grilling, roasting, and deep-frying, enhance the flavor of food and the desired texture in the final cooked product. This is attributable to a unique chemical reaction known as the Maillard reaction [1]. This reaction was named after the French scientist Louis Camille Maillard who attempted protein synthesis in his laboratory in 1912, but the reaction mixture ended up creating a faux meaty aroma and flavor [1,2,3]. These byproducts are the most extensively studied group of heterogeneous molecules that are formed in vivo while creating additional food flavors [1]. Reducing sugars (glucose, galactose, and fructose) readily bind with the amino group of free lysine, arginine, and sometimes cysteine, tryptophan, and histidine, resulting in the formation of the Maillard reaction glycated products. This occurs under normal physiological conditions through a series of nonenzymatic reactions [4,5], as presented in Figure 1.

**Figure 2:**
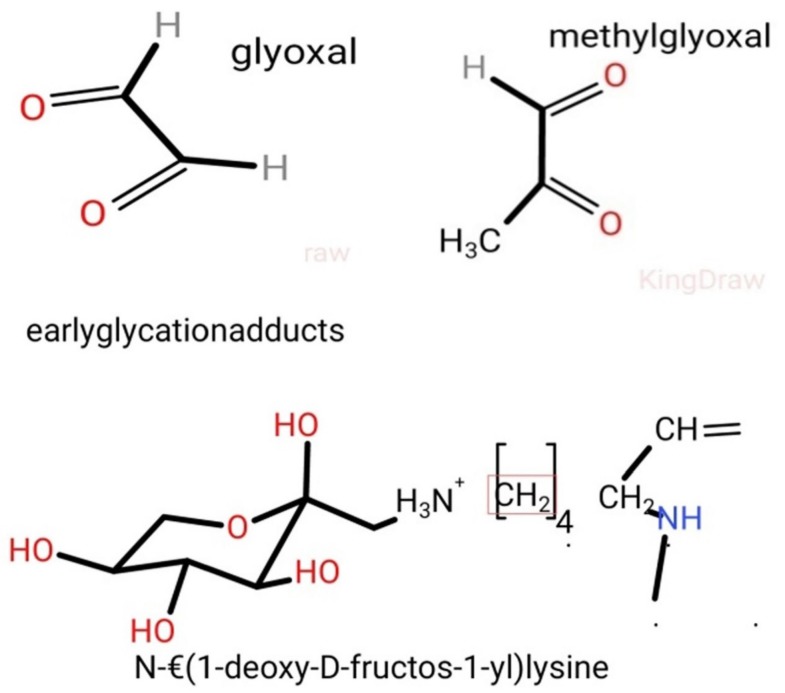
Early glycosylation protein adducts, such as glyoxal, methylglyoxal, N-ε(1-deoxy-D-fructose-1-yl)lysine. (Diagrams are drawn using the KingDraw application software).

These intermediate products are known as Schiff’s bases or Amadori products, which are chemically reversible molecules formed endogenously. These products undergo conformational changes (such as rearrangement of the molecules), which stabilizes them into the final heterogeneous products, abbreviated as AGEs (Figure 2). Following their conformational changes, the products bind tightly with proteins available in the vicinity [6] and further crosslink with long-lived proteins, such as collagen, lens protein [4], hemoglobin [7], lysozyme, alkaline phosphatase, elastin, etc. [8]. These proteins are present in low abundance [9] in almost every tissue and generally produce a temporary or reversible effect during binding [10].

Glycation of some important proteins, such as β-amyloid present in neuronal tissue, can affect the progression of Alzheimer’s disease (AD). Their accumulation in brain tissues represent a marker for the diagnosis and prognosis of the disease by forming amyloid plaques, neurofibrillary tangles, and activated microglia [11,12,13]. With the involvement of structural proteins, such as collagen, the in vivo crosslinking of AGEs results in the hardening of the extracellular matrix, causing further dysfunction of vessels and organs [7].

Various detection and measurement databases [9,14] prove that AGEs are formed in both processed and unprocessed foods [5]. Endocrine disruptors are a group of chemicals present either naturally or synthetically in foods and various substances. These disruptors have a great affinity towards hormones, are detrimental to the endocrine system, and affect the overall health. Presence of these chemicals in food is increasing as a result of modern inventions in food science and technology [15,16]. One of the widely used food products is infant formula milk, processed in huge mechanical plants at high temperatures. Absorption of formula milk elevates the serum levels of AGEs at a very early age [17]. 

## 2. Advanced Glycation End Products (AGEs) and Modern Diet 

There are two types of AGEs: endogenous and exogenous. The Maillard reaction is a common nonenzymatic reaction that usually occurs after assimilation of food. Biomolecules are readily available to react with the pool of Maillard products within the body, which consists of both exo- and endogenous AGEs. Long-lasting proteins, such as collagen, that come into contact with free circulatory glucose contribute towards endogenous AGEs. Exogenous AGEs comprise cooked or processed foods, beverages, and other food items. Exogenous AGEs contribute more towards the AGE pool than endogenous AGEs [2,18,19,20].

Thus, it is important to analyze and determine the content and amount of AGE in a food product. Examining the association between serum AGE and RAGE (receptor for AGEs) may help to understand the underlying causes of specific health issues [15].

Some of the approaches to analyze dietary AGEs are as follows:

- Enzyme-linked immunosorbent assay (ELISA)-based immunochemical detection [9,21];
- High-performance liquid chromatography (HPLC) [22];
- Fluorescence detection, as some AGEs emit characteristic fluorescence [9];
- Mass spectrometry;
- Gas chromatography with mass spectrometry [23];
- MALDI-TOF mass spectrometry [24];
- Western or dot-blot assays [18].

## 3. AGEs: Formation and Absorption

**Figure 3:**
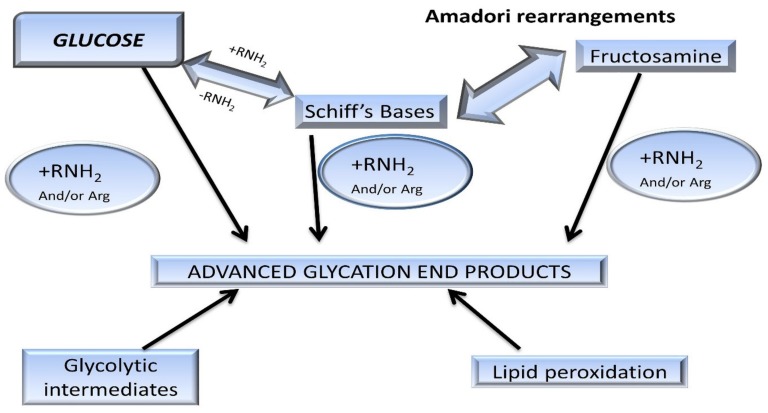
Pathway of protein glycation forming Maillard reaction products (MRP). Amadori rearrangements are highlighted within the early reversible adducts, such as Schiff’s bases. These glycated adducts are irreversibly modified into advanced glycation end-products [14].

Chemically, AGEs are formed by the condensation of an aldehyde group of sugar with amino groups of protein, resulting in the formation of reversible products known as Schiff’s bases [5], such as aldimine. These products are maintained at an alkaline pH, usually greater than 7. The formation of AGEs is promoted at higher pH. The rearrangement of aldimines depends on the number of free sugar/amino groups attached or available along with the pH being maintained above 7 [7]. This is also known as the initial phase. The aldimines further rearrange into covalently-bound Amadori products [3] (an important milestone in the glycation of proteins). Schiff’s bases (Figure 3) specifically rearrange themselves when the pH drops below 7. This phase is also known as the proliferation stage. Foods below the pH of blood might be responsible for the pathology of various diseases [7]. All products are reversible up to this stage. The numerous rearrangements of the Amadori products, such as isomerization, epimerization, and hydrogen bonding, result in the formation of final irreversible molecules known as AGEs. This stage is known as the advanced proliferation phase of glycation [9,25,26].

In our biological system, there are various types of active AGE molecules exerting different physiological effects [4,18].

Commonly found AGEs in foods are listed below [14]:

- CML: N_ε_-carboxymethyl-lysine;
- CEL: N_ε_-1-carboxyethyl-lysine;
- Pyrraline;
- Glyoxal;
- Methylglyoxal;
- Acrylamide;
- Furan [27];
- Derivatives of bis(lysyl)imidazolium:
  - DOLD: Deoxyglucosone-derived lysine dimer[1,3-di(Nε-lysino)-4(2,3,4-trihydroxybutyl)-imidazolium salt]
  - GOLD: glyoxal-derived lysine dimer[1,3-di(N^ε^-lysino) imidazolium salt].

Only 10–30% of exogenous AGEs are absorbed into the systemic circulation when administered orally [28]. The maximum absorbed dietary AGEs are pyrraline and pentosidine (around 60–80%) [4]. Most of these absorbed AGEs are free single amino acids, low molecular weight peptides, or high molecular weight compounds [29]. CML is usually absorbed by simple diffusion [30], whereas pyrraline is absorbed in the intestinal epithelium as a dipeptide via peptide transport [31]. Proteolytic digestion is the primary step in the digestion of proteins; however, modification of proteins by AGEs results in hindered absorption and digestion of proteins [29,32]. Post digestion, fructose-lysine and CML are bound to peptides smaller than 1000 Da, making them readily available for absorption [33]. Maillard reaction products further reduce the uptake of regular proteins via the epithelium; this crosslinking promotes uptake via Peyer’s patches, e.g., β-lactoglobulin and α-lactalbumin. The in vivo absorption and assimilation of CEL and CML were studied in rats [34] by intravenous administration of these glycated products. It was observed that these products target and temporarily accumulate in the liver. To assess the distribution and assimilation, radioactive studies on AGEs revealed that, after 72 h of exposure, 60% of the radioactivity is detected in the liver and kidneys, as well as in the lungs, spleen, and heart [35].

## 4. Food Processing and AGEs

Some cooking techniques have been adapted from our ancestors and some have developed because of increased demands due to an expanding world population. The stressful and continuous workaholic environment has directed people towards pre-prepared and processed foods, which, although delectable to the palate, possess the highest amount of exogenous AGEs compared to freshly home-cooked meals. Data indicate that foods believed to be remedial are in fact sources of inflammation and causative agents in the development of numerous diseases. These could be attributed to advancements in food science and technology [36,37,38].

Most AGEs are produced in foods processed with dry heat technology. This might be the reason that convenient food items, such as cookies, biscuits, and chips, have high amounts of AGEs since they contain one or more AGE-producing ingredients, such as cheese, nuts, fats (saturated/trans), and butter. Today, a large number of consumers of these products are toddlers and young children who are below 15 years of age. This is an alarming sign, as an increasing exogenous AGE load makes them more prone to adverse outcomes, such as diabetes, obesity, cardiac diseases, renal failure, or even dementia [15,18,19,36,39,40].

Cai et al. investigated the role of dietary AGEs in dementia using the mouse model and proposed that dementia related to age can be linked to food AGEs in their diet, specifically methyl-glyoxal [41]. Intake of AGEs has also been associated with the onset and complication of many diseases, such as diabetes, cardiovascular diseases, and neurological disorders [39,42,43,44,45,46,47].

Various databases have revealed the presence of AGEs in a broad range of food products, such as biscuits, bread, cheese, peanut butter, and processed meats. [38]. The main problem is that the most commonly formed glycation products, such as CML or CEL, are partial products of carbohydrates already present in the food. The highest amount of CML/CEL ranges from 5 to 7 mg/100 g food, such as in peanut butter and chocolate sprinkles [24], and in high-temperature cooked food items, such as fried potatoes and grilled chicken, it may range between 5 and 500 µg/100 g food [21] (Table 1). Glyoxal, methyl glyoxal, and 3-deoxyglucosone are byproducts of bakery foods, such as bread and biscuits, and are also prominently found in carbonated drinks, containing high fructose corn syrup, and in some fermented products, such as wine and beer [48,49].

## 5. Exposure of Infants and Toddlers to Exogenous AGEs

**Figure 4:**
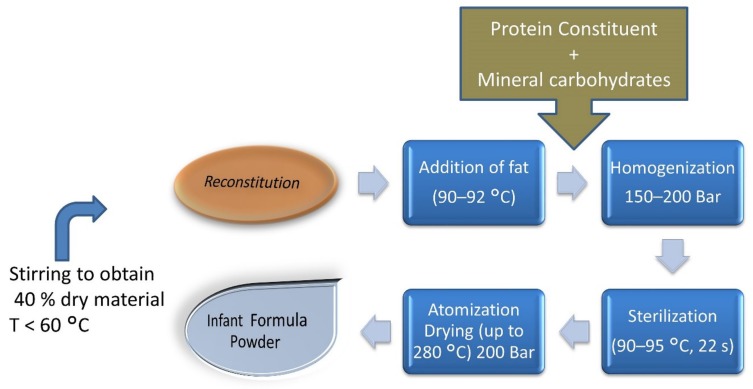
Formulation of infant formula milk powder describing high-temperature ranges during its complete process. Reconstitution of 40% dry material obtained by stirring at T < 60 °C, and high-temperature processing at later stages, i.e., addition of fat at 90–92 °C, sterilization at 90–95 °C, and atomization drying up to 280 °C [27].

The most unfortunate event is the exposure of infants and toddlers to exogenous dietary AGEs, as early as days to weeks after birth. Studies have shown that the dry heating of milk elevates dietary AGEs or glycation markers in infant formula milk compared to regular cow milk products. As milk sugar (lactose) and its protein (whey) are readily available, and subsequent heating over higher temperatures during processing to achieve the desired product results in the creation of Schiff’s bases or Amadori adducts in formula milk (Figure 4).

During the gestational period, even a mother is exposed to numerous exogenous glycated proteins that eventually pass to the infant through lactation. Kutlu T. (2016) described that infants can receive up to 15 kU/kg AGEs directly from breast milk, which might increase to 76 kU/kg until they reach the age of 6 months. The major glycotoxin found in infant milk is CML, with levels of up to 1.3–1.5 mg/kg in babies fed formula milk containing high levels of CML and reaching as high as 160–630 ng CML/mg protein [17,53]. Prosser et al. found very high quantities of CML in infant formula. Goat milk formula was determined to have 7- to 12-fold less CML when compared to cow’s milk [54].

## 6. AGEs: Health and Disease

AGEs exert two types of effects: receptor-independent with crosslink formation and RAGE-linked receptor-dependent. Exogenous AGEs bind to its receptors, inducing transcellular and endogenous pathways, which further modify signaling molecules, such as NF-Kβ and STAT3. Functions of only a few receptors and binding proteins for AGEs have been identified, which include RAGE, AGE-R1, R2, and R3, and scavenger receptors. These receptors are present on hematopoietic, neuronal-glial, renal, and vascular cells [27].

It is well established that protein glycation is a regular chemical reaction; the formation of glycated protein and its stability are dependent on the protein it reacts with and the tendency of the body to break it back into its constituent products utilizing normal methods of detoxification under aerobic and anaerobic conditions. With disease (whether pathogenic or pathological) progression in the body, the detoxification processes either slow down or tend to become impaired, causing these molecules to accumulate or aggregate within the body as they may not be flushed out of the systemic circulation [18,25].

Not all, but most of these diseases, are grouped as metabolic disorders, including obesity, diabetes, hypertension, cardiovascular disease, hyperlipidemia, and hypercholesterolemia [55], as well as cancers of various tissues or endocrine diseases, such as hypothyroidism and hypoparathyroidism [56]. These diseases result from metabolic imbalance or the inability to maintain glucose homeostasis. Any imbalance due to substrates, such as fats, carbohydrates, or proteins, results in the disruption of various molecules, metabolites, and their pathways or systems. For example, glycogen-storage disease type 0 due to a deficiency of glycogen synthase enzyme causes hypoglycemia, ketosis, and early child death; McArdle’s disease due to a deficiency of muscle phosphorylase enzyme causes poor muscle exercise tolerance or easy fatigability; protein-related disorders, such as Parkinson’s disease, due to dopamine deficiency; albinism due to melanin deficiency; and congenital abnormalities, such as Crigler–Najjar syndrome [18,19,36,43,57]. Although AGEs influence a number of organs and their functions, we discuss only a few diseases wherein the glucose influx and its metabolism are responsible for the increase in endogenous AGEs. A description of organs/diseases is given below, which proves that exogenous AGEs are responsible for their pathophysiology (Table 2).

### 6.1. AGEs, Diabetes, and Their Related Disorders

As carbohydrates are the preferred food source and are consumed in abundance along with dietary proteins (the building blocks), a question arises about why this post-translational modification of proteins (glycation) is lethal to the body [9]. The most common metabolic disorder is diabetes and its global prevalence rate has risen from 4.7% to 8.5% from 1980 to 2014, among adults over 18 years of age (World Health Organization), affecting 422 million people in 2014 and increasing daily [18,70]. It is apparent that the formation of AGEs is consistently high due to hyperglycemia in diabetes. The observation of high levels of skin collagen crosslinked AGEs is an indicator of diabetes type 1 along with carotid thickening. In Diabetes mellitus type -2, AGE levels can be potential markers that directly correlate the disease with hypertension and ischemic heart disease. 

Sell et al. showed that increased crosslinking of collagen with pentose sugars, such as ribose, contributes to advanced glycation in diabetes and end-stage renal (ESR) disease. This was also observed in diabetic patients with ESR requiring hemodialysis and/ or renal transplant. The author concluded that abnormalities in pentose sugar metabolism and its crosslinking with heavy proteins, such as collagen, augment the consequences of microangiopathy, cardiovascular disease, cataract, or basement membrane thickening, and other conditions [70]. Dozio et al. found an association of soluble receptor for AGEs (sRAGE), fibroblast growth factor 23 (FGF-23), and cardiovascular complications in diabetic chronic kidney disease patients. Glycated albumin levels were found to be higher in the DM group when compared to the non-DM group. A study by the Diabetes Control and Complication Trial proved that serum levels of AGEs are better predictors of diabetes compared to levels of HbA1C, which is also related to renal or hepatic impairments [77]. sRAGE levels were higher than the normal range in both the DM and non-DM group. However, since the DM group ranges were statistically higher than the non-DM group, the author proposed that sRAGE can be a marker for cardiac remodeling [78].

Diabetic retinopathy has been a leading cause of blindness in the past two decades. The formation of endogenous AGEs is associated with microvascular complications in patients suffering from diabetes. Increased levels in the serum and vitreous fluid of eyes of diabetic patients is considered to be a marker for early detection of diabetic retinopathy [79,80]. Elevated levels of AGEs have also been detected in peripheral nerves of the optic area [79].

Normand et al. performed a pilot study designed to understand renal performances of healthy participants administered high-AGE protein diets for five months. Renal perfusion assessed (by PET) after meals containing high AGEs revealed significant increases. Oxidative stress was also slightly higher from baseline levels [81]. Inhibition of the AGE-RAGE complex by antagonists, such as linagliptin, proved to have a potent protective mechanism from renal damage in diabetes [82]. Diabetes is known to cause vascular permeability and vascular dysfunction was detected on day 10 of exogenous administration of AGEs, which persisted up to 4 weeks. The author therefore concluded that circulatory AGEs are important “uremic toxins” responsible for progressive vascular diseases, such as uremic cardiomyopathy [64]. In diabetic patients with ESR disease, they may result in rapid clinical deterioration.

Crosslinking of AGEs contributes to the rigidity of arterial and connective tissues and increases when a person is hyperglycemic. The role of AGEs in the initiation and progression of atherosclerosis via modification of lipoproteins is proven; its crosslinking ability facilitates aggregation of basophilic degeneration deposits in myocardial fibers, which are insoluble in nature [83].

Glycation of proteins may lead to macrophagic-monocytic chemotaxis [63]. Inactivation of nitric oxide further lowers the effects of nitric oxide, a key compound in vasodilation [84] and endothelial-derived relaxing factor. These glycated products alter both the phospholipid and apo-protein components of low-density lipoproteins [85], which are found to be 2- to 4-fold more in patients with diabetes.

### 6.2. AGEs and Brain Disorders

The brain is the third largest organ in the body, weighing around 1380 g, and consumes most of the glucose as a primary food source. At physiological pH, chances of glycation in the brain are higher compared to other organs. In contrast, patients suffering from disorders, such as Alzheimer’s disease AD or another amyloidosis, have plaques that represent *β* amyloid protein (*β*AP) aggregates, which are typically neurofibrillary tangles or cerebrovascular amyloid deposits. Reports indicate that RAGE knockout mice have less plaques showing an advanced stage of amyloidosis. A protein called A-ApoA-II protein depositions were in the range 1.1 ± 0.2 A.U., which were lower in RAGE-l-, compared to wild types having <2.5 ± 1.3 A.U (*p* < 0.001) [86]. Protein aggregates of AD plaques are modified due to advanced glycation, which are found to be 3-fold higher when compared to healthy controls. AGE-modified *β*AP stimulates their aggregation as plaques in AD and other neurodegenerative diseases and further decreases the cognitive abilities of patients [87]. Some scholars believe that there is a high association of diabetes with AD and suggest that AD should be considered another type of diabetes mellitus.

### 6.3. AGEs and Women’s Health

A woman’s ability to reproduce is inevitable in nature. The Darwinian evolutionary theory describes “survival of the fittest”, but the meaning of survival has now modified to mean better education, a stressful workplace, and an exhausted household due to increasing competition. A woman’s health is compromised at every step. Working women are more affected as they are required to balance between their home and office life. They are at a higher risk of stress, indigestion, and peer pressure to drink and smoke, which altogether degrades health.

**Figure 5:**
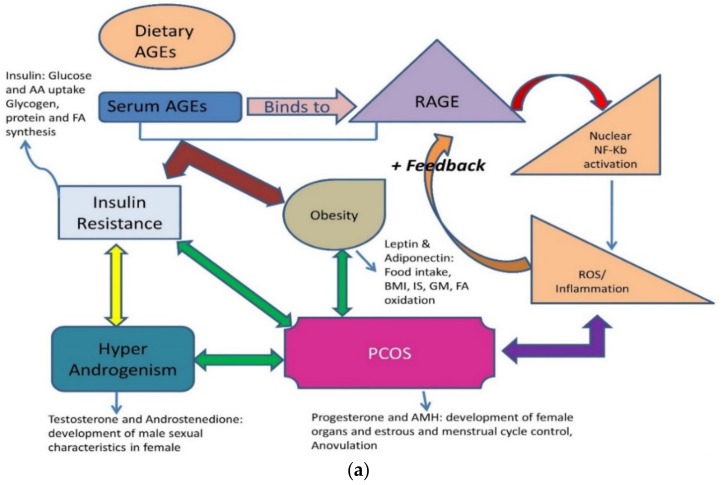

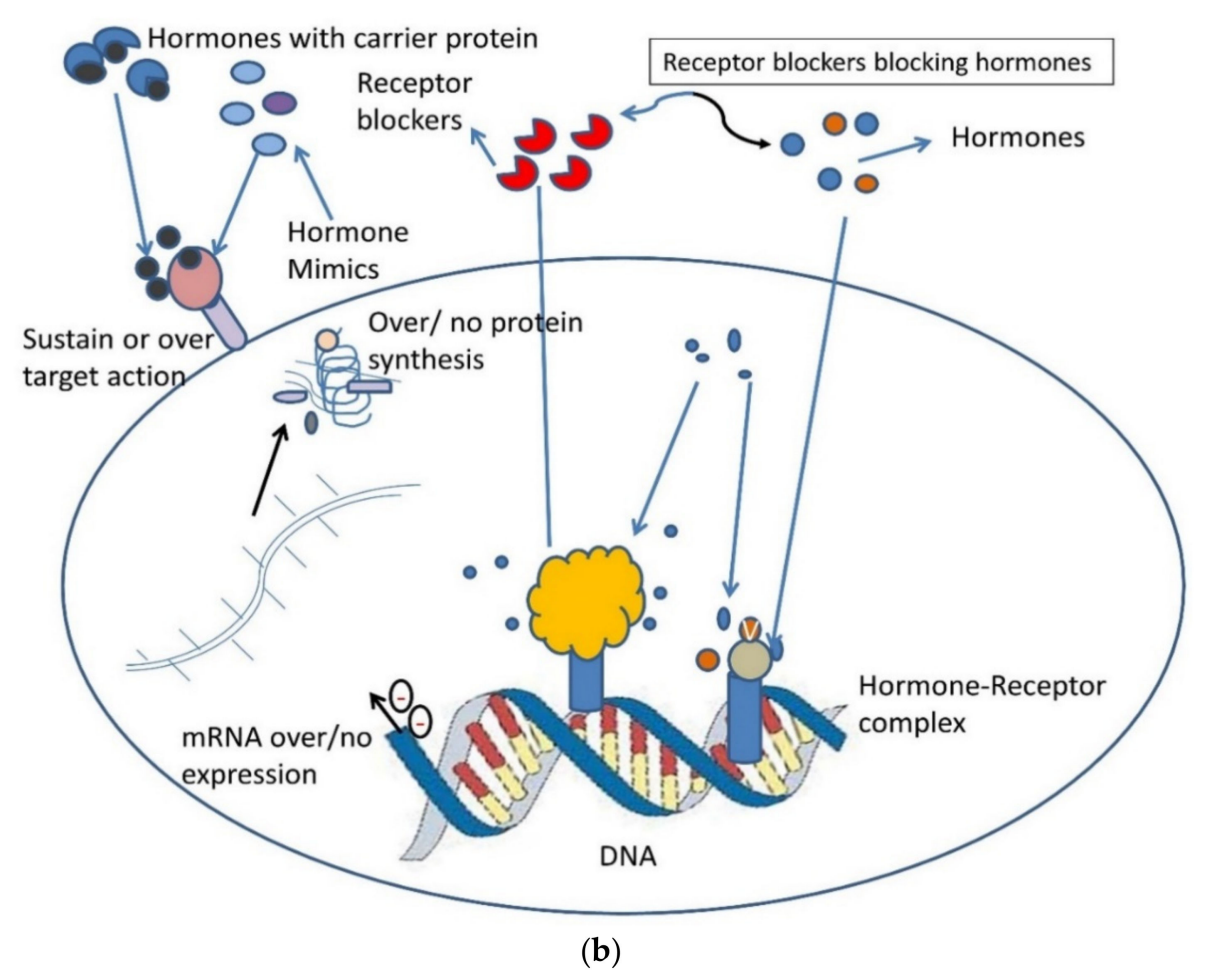
(**a**) An illustration of the role of dietary advanced glycation end products and common lifestyle disorders. Graphic interpretation of the relationship of diseases with serum levels of AGEs and RAGE [15,90]. RAGE: Receptor for Advanced glycation end products; PCOS: polycystic ovarian syndrome; ROS: Reactive oxygen species; BMI: Body mass index; IS: Insulin Sensitivity; GM: Glucose metabolism; AA: amino acid; FA oxidation: fatty acid oxidation. (**b**) Pictorial representation of the molecular hacking of hormones and their receptor complex by AGEs: Binding of AGEs to a hormone receptor by mimicking it or trapping of hormones by blocking their receptors. AGEs can bind antagonistically to cell surface receptors causing over/no protein expression [15].

Obesity, DM-2, thyroid disorders, infertility, and PCOS are the most common lifestyle diseases. They are interconnected, with one affliction leading to another. Menarche age has reduced to 11–13 years, indicating that girls are attaining puberty at a very early age and their enhanced growth shows an increased stimulus to endocrine glands externally by the environment and internally by stress and diet. Both endogenous and exogenous AGEs are responsible for disrupting the functioning of various endocrine glands and their hormones (Figure 5a,b). The reasons are multi-factorial; clinicians face the dilemma of considering whether PCOS is a metabolic, reproductive, or endocrine disorder. These ovarian dysfunctions can be a collective of all disorders and can be summed up as an interrelated disease [88,89,90,91,92,93] (Table 3).

A study between two food groups was done comparing (i) low AGEs, foods prepared at low temperature, and (ii) high AGEs, foods cooked at high temperatures, such as grilling, frying. The expandable data obtained revealed that women that consume foods cooked at higher temperatures and with high AGE contents had significantly higher levels of total testosterone, insulin, androstenedione, and irregular menstrual cycles, relating them to ovarian dysfunction [89].

Estrogen and progesterone are important hormones for implantation of embryo in the uterus. A study on Wistar rats having highly expressed RAGE in mononuclear cells (peripheral blood) revealed low levels of the hormones, along with high levels of glucose, insulin, and testosterone. This data verified that PCOS is an endocrine disorder that is highly influenced by metabolism and diet [71,92,108]. Increased levels of anti-Mullerian hormones (AMH) have a sustainable effect on the reproductive system by provoking anovulation via direct inhibition of FSH. Women habituated to high consumption of AGEs had elevated levels of testosterone, AMH, and androgens [92,109].

Another study reported the association of a high AGE isocaloric diet (HA) with PCOS in women. Women consuming a high HA diet had elevated levels of serum AGEs, testosterone, insulin, and oxidative stress, as compared to women on low AGE isocaloric diet. Dietary changes may contribute to alterations in PCOS and its symptoms, but are independent of anthropometric parameters [91]. Protein levels revealed an upregulation of RAGE in the circulatory monocyte, along with high levels of serum AGEs in women suffering from PCOS, who were insulin resistant but not hyperglycemic. The interaction of AGE-RAGE also exhibits a critical role in down-regulating the signal cascade, thus altering steroidogenesis in the reproductive organs of women with PCOS [90,108]. Lysyl oxidase also interacts with AGE signaling to modulate collagen synthesis in PCOS [98]. Renin-angiotensin-aldosterone system has been reported to disrupt cardiovascular functioning, causing hypertension in women with PCOS [110].

Vitamin D (cholecalciferol) has a direct link in controlling and maintaining the pathophysiology of PCOS and ovarian dysfunction. Vitamin D supplementation in patients ameliorates the effects of AGEs in PCOS [37,111]. In a group of women afflicted with PCOS and vitamin D3 deficiency, and subsequently treated with supplementation, levels of AMH were decreased and sRAGE were significantly increased compared to untreated controls (no vitamin D3 supplementation). Vitamin D3 also executes the mRNA expression of genes responsible for steroidogenesis [108], such as *CYP11A1, CYP17A1, CYP19A1, HSD, LHR, FSHR*, etc. The mRNA expression of these genes can be modified by human glycated albumin (HGA) with or without vitamin D3. Granulosa cells on HGA treatment without vitamin D3 showed increased expressions of CYP11A1 (48%), 3-βHSD (38%), StAR (42%), CYP17A1 (30%), and LHR (37%) without affecting the levels of CYP19A1 and FSHR. In addition to vitamin D3 with HGA, there was a significant decrease in all gene mRNA expressions, with the exception of 3-βHSD which remained as high as 48% when compared to controls [106,108].

Women with PCOS usually have low Vitamin D3. However, the vitamin is required for the sustainable functioning of ovarian follicles and oocyte development [81,82,83]. Vitamin D3 not only down-regulates the AGE-RAGE interaction, but also attenuates overexpression of RAGE mRNA, as AGE mimics hormones and disrupts its expression (Figure 5a), making it an important parameter to understand the pathophysiology of PCOS [88,98,111,112,113,114].

### 6.4. AGEs and Allergies

AGE-induced immunotoxicity activates cytokines and impedes the functioning of NLRP (nucleotide-binding oligomerization domain, leucine-rich repeat, and pyrin domain-containing) family and toll-like receptors. Alarmins are unique biomolecules that are normally secreted due to immune responses by cells following unprogrammed cell death. Major alarmins, such as high mobility group box 1 (HMGB1), are secreted by dendritic cells and help in the proliferation and activation of T-lymphocytes [75]. This HMGB1 binds efficiently with RAGE and further helps in the activation and migration of various immune responding cells, such as monocytes, macrophages, neutrophils, and dendritic cells. RAGE also activates other alarmins, such as S100 protein and β amyloid peptide [114,115]. AGE exposure may increase the risk of auto-immune diseases, such as asthma, multiple sclerosis, and Crohn’s disease, with a comparable decreasing pattern in infectious diseases. High levels of AGEs are found in processed foods in western diet outlets such as McDonald’s and Kentucky Fried Chicken (KFC) [75,76,114].

### 6.5. AGEs and Dental Disorder

Teeth and other lingual tissues are exposed to food before it leaves the mouth and enters the digestive tract. A study of the gingival crevicular fluid in DM patients with chronic periodontitis revealed increased levels of AGEs, suggesting the requirement of acute dental care in DM patients. AGE levels were significantly higher when compared to systemically healthy individuals with and without periodontal diseases [116].

## 7. Conclusions

Maillard reaction, commonly known as protein glycation, normally occurs in vivo as well as during the preparation of foods at high temperatures. Though it is a simple reaction, elevated serum levels of AGE are risk factors associated with the physiology of various metabolic disorders. Formula milk is a rich source of AGEs and its consumption makes infants more prone to inflammation, secondary infections, or auto-immune diseases.

Studying AGEs will help us in better understanding the glucose metabolism. Impaired glucose metabolism may be the underlying reason for numerous diseases and may link the pathways that are still untouched by the scientific world. Food, which is important to survive, has in recent years become a reason for many diseases. Researchers with their numerous studies and skills have determined what types of food and cooking techniques contribute to the severity of diseases, such as dementia, chronic renal failure, DM-2, retinopathy, nephropathy, dental health, and allergies. AGE reservoirs are overflowing in vivo, making it possible for us to heal naturally. Exploring these parameters has opened gates to search loopholes for future research. (Table 4).

## 8. Limitations

- Our review is limited to the biochemical basis of AGEs. However, serum AGEs are regulated by a receptor-mediated pathway known as the AGE-RAGE system; this mechanism gives us a broader view of their functioning. Glycation is a post-translational modification and requires an understanding of the genetic basis of this modification, efficacy of advanced glycation in the molecular basis of disease, and persistence of certain diseases. Therapeutic interventions to reduce the effects of advanced glycation in maintaining the pathophysiology of diseases, such as AD, dementia, diabetes, PCOS, ESR, aging, etc., were not covered by this review.
- Role of AGEs in oxidative stress is a limiting factor of this review
- We have described limited knowledge to understand the relationship between PCOS and AGEs. Deeper understanding can reveal newer aspects of PCOS that later manifest as infertility, thereby degrading a woman’s health.

## Figures and Tables

**Table 1:** Commonly found exogenous advanced glycation end products measured via HPLC and ELISA and their approximate range found in consumables

Maillard Reaction Product	Type of Foods	Food Processing	Advanced Glycation End Products Range in Different Foods	References
Acrylamide (a by-product of MRP) [27]	Starchy, potato-based foods, such as french fries.	Frying Grilling Baking	Fried potatoes: 272–570 µg/kg^−1^ Bakery products: 75–1044 µg/kg^−1^ Breakfast cereals: 149 µg/kg^−1^	[50]
Furan [27]	PUFA-rich foods, carotenoids, or vitamin-containing foods.	Roasting Frying Caramelizing Pasteurization	Espresso coffee: 936 ng/g^−1^ Potato chips: 259 ng/g^−1^ Jarred baby foods: 8.5 ng/g^−1^ Orange juice: 7.0 ng/g^−1^	[51]
CML [4]	Infant formula, milk and dairy products, boiled eggs, peanut butter, beef, chicken, meat.	Roasting Charring Boiling Baking Grilling Toasting	Peanut butter, chocolate sprinklers: 5–7 mg/100 g protein Milk chocolates: 0.01 mg/100 g protein Milk samples: 2.7 mg/100 g protein White bread, boiled eggs: 11.2 mg/100 g protein Grilled chicken: 5–500 µ/100 g product	[4,24]
CEL [4]	Sponge cakes, potato chips, peanut butter.	Baking Roasting Frying	Deep-sea fish: 2.49–249 ng/mL Peanut butter: 7 mg/100 g product	[4]
Methyl glyoxal, glyoxal, 3-deoxyglucosone [4]	High-fructose corn syrup	Pasteurization Boiling Baking High-pressure processing	Fruit juices: 410 mg/L Balsamic vinegar: 2622 mg/L Cookies: 385 mg/kg Carbonated soft drinks: 0.3–1 mg/L	[48,49,52]

(CML: Nε-carboxymethyl-lysine; CEL: Nε-1-carboxyethyl-lysine; PUFA: polyunsaturated fatty acids).

**Table 2:** Various effects of AGEs on different organs and their specific proteins or diseases

Organ/Disease	Binds to	Effects
Brain	Amyloid protein	Increases β-amyloid plaques [58], resulting in dementia [59,60] or severity in schizophrenia [61]
Skin	Articular collagen, skeletal and smooth vascular muscles, glomerular basement membrane	Reduces flexibility, alterations of co-functions, such as migration, differentiation, and proliferation [62,63]
Kidney	Bowman’s capsule	Accumulation of uremic toxins [64], the appearance of complications, such as poly-nephropathy [8], chronic renal failure [65]
Eyes	Opsin	Macular degeneration of the retina [66,67]
Heart	Vessels	Progression of coronary heart disease or myocardial damage [68]
Photoaging	Fibroblasts/keratinocytes Superoxide dismutase	Cells become more sensitive to exposure to UVA radiations and their viability decreases, impairing repair mechanism [62,69]. Compromise cellular antioxidant defense system [69]
Joints, lungs, heart, skin, blood or combination of these, Systemic Lupus Erythematosus	White blood cells	Inflammation in mentioned organs attacking own cells, face rashes, flare, sensitivity to light, swelling, etc. [7,69,70,71]
Diabetes	Low-density lipoprotein	During chronic hyperglycemia, promotes the initiation of lipid peroxidation in vivo [3,62,72] Macro and microvascular complications of diabetes [73,74,75,76,77]

**Table 3:** Tabular representation correlating AGEs and its effects in PCOS

Biochemical Changes	Effects	Ref.
High levels of testosterone and androstenedione	Irregular menstrual cycles	[94,95,96]
Raised expression of Receptor of AGE (RAGE) in mononuclear cells along with increased glucose, insulin, and testosterone	PCOS characterized as both endocrine metabolic disorders	[71,95,97]
High AGE diet elevates anti-Müllerian hormone, inhibits Follicle-stimulating hormone	Provokes anovulation	[98,99]
High AGEs isocaloric diet elevates testosterone, insulin, and oxidative stress contributing to PCOS and its symptoms	Irregular menstrual cycles, high ovarian cysts	[92,97,100]
Upregulation of RAGE in PCOS, downregulation signal cascade of steroidogenesis in women of reproductive age	Disruptive hormone formation	[91,101]
Vitamin D3 supplementations reduce the effects of AGEs in PCOS	Attenuates AGEs and supports ovarian health	[102,103]
Excess deposition of collagen	Cyst formation in ovaries due to enzyme lysyl oxidase	[102,104,105]
Disruption of renin-angiotensin-aldosterone system	Disturbed cardiovascular functioning and hypertension	[37,106,107]

**Table 4:** Summary of the deleterious effects of glycated proteins in the pathophysiology of certain metabolic disorders

Disease	Effects of AGEs	References
Diabetes	Crosslinking of skin collagen, carotid thickening, ischemic heart attack, chronic and end-stage renal disease, diabetic retinopathy, uremic cardiomyopathy, alterations in lipo- and apolipoproteins, inactivation of nitric oxide	[64,70,71,72,73,74,75,76,77,78,79,80,81,82,83]
Alzheimer’s disease, certain neurodegenerative diseases, advance stages of amyloidosis	β-amyloid protein plaques, cerebrovascular amyloid deposits, neurofibrillary tangles	[85,86,87]
Ovarian dysfunction, polycystic ovarian syndrome, anovulation, infertility	Increased testosterone, thyroid hormones, androgens, anti-Mullerian hormone, disruptive steroidogenesis	[71,92,108]
Inflammation, allergies, asthma, multiple sclerosis, Crohn’s disease	Activating unprogrammed cell death, altering immune responses by monocytes, basophils, macrophages, and dendritic cells, might create false allergic responses	[75,76,114,115]
Dental disorders	Periodontitis leading to tooth loss due to gum infection might increase the risk of heart and lung diseases	[116]

## Abbreviations

AGEs	Advanced Glycation End Products
AMH	Anti-Müllerian hormone
PCOS	Polycystic ovary syndrome
AD	Alzheimer’s disease
DM	Diabetes mellitus
CML	Carboxy-methyl lysine
CEL	Carboxy-ethyl lysine
